# Modulation of thymidine phosphorylase by neoadjuvant chemotherapy in primary breast cancer

**DOI:** 10.1038/sj.bjc.6601845

**Published:** 2004-05-04

**Authors:** M Toi, H Bando, S Horiguchi, M Takada, A Kataoka, T Ueno, S Saji, M Muta, N Funata, S Ohno

**Affiliations:** 1 Department of Surgery, Tokyo Metropolitan Komagome Hospital, 3-18-22, Honkomagome, Bunkyo-ku, Tokyo 113-8677, Japan; 2 Department of Breast Surgery, National Kyushu Cancer Center, 3-1-1, Notame, Minami-ku, Fukuoka 811-1395, Japan; 3 Cancer Center Karolinska, Department of Oncology and Pathology, Karolinska Institute and Hospital, Stockholm S-171 76, Sweden; 4 Department of Pathology, Tokyo Metropolitan Komagome Hospital, 3-18-22, Honkomagome, Bunkyo-ku, Tokyo 113-8677, Japan;

**Keywords:** TP, preoperative chemotherapy, fluorouracil, immunohistochemistry, upregulation

## Abstract

The combination effect of docetaxel and capecitabine on tumour response rate and survival was demonstrated recently in metastatic breast cancer patients. This combination was based on an experimental hypothesis that taxane can increase tumour sensitivity to the effect of capecitabine through the upregulation of thymidine phosphorylase (TP), which is responsible for the metabolism of 5-fluorouracil (5-FU) and its derivatives, including capecitabine. To examine the alteration in TP expression before and after neoadjuvant chemotherapy, 92 patients with primary breast cancer (T2-4N0-1M0) were enrolled in this study; 14 were treated with adriamycin and cyclophosphamide (AC) or epirubicin and cyclophosphamide (EC); 58 with 5-FU, adriamycin, and cyclophosphamide (FAC) or 5-FU, epirubicin, and cyclophosphamide (FEC); and 20 with FEC followed by docetaxel/taxotere (TXT-containing regimen). Thymidine phosphorylase upregulation was seen in 54.4% and 32.6% of patients in tumour cells and stromal cells, respectively. Increases in TP expression were found only in the AC/EC and TXT-containing regimen groups. In conclusion, it was strongly suggested that unlike 5-FU-containing regimens, the taxane and AC combination therapies upregulate TP expression in primary breast cancer. Thymidine phosphorylase upregulation by several anticancer drugs implies the importance of individualised strategies for sensitisation of tumour tissues to 5-FU and its derivatives.

Thymidine phosphorylase (TP) is an enzyme that is responsible for nucleoside metabolism, antiapoptosis activity, and promotion of angiogenesis. Thymidine phosphorylase acts mainly in the salvage cascade of DNA metabolism in response to various types of stresses. Thymidine phosphorylase functions in the prevention of hypoxia-induced apoptosis according to recent experimental analyses (Ikeda *et al*, 2003). In addition, it has been documented that a metabolite of thymidine generated by TP, 2-deoxy-D-ribose (2-DDR), acts as a potent chemotactic factor on the endothelium, which results in the promotion of neovascularisation (Haraguchi *et al*, 1994). In fact, in a variety of tumour tissues, overexpression of TP was found to correlate significantly with an increase in neovascularisation (Toi *et al*, 1995; Tanigawa *et al*, 1996; Matsuura *et al*, 1999) and poor prognosis (Maeda *et al*, 1996; Takebayashi *et al*, 1996; Koukourakis *et al*, 1998; Toi *et al*, 1999).

The regulation of TP has been also studied from various points of view. Generally, TP is upregulated by stress such as hypoxia (Griffiths *et al*, 1997), radiation (Sawada *et al*, 1999), and chemotherapeutic damage (Sawada *et al*, 1998; Endo *et al*, 1999). Several types of cytokines such as interleukin (IL)-1, tumour necrosis factor (TNF)-*α*, and interferon (IFN)-*γ* also upregulate the expression of TP in both nonmalignant and malignant cells (Eda *et al*, 1993). Therefore, it is likely that these factors have important functions in stress-induced TP upregulation.

Thymidine phosphorylase has also been studied as a key enzyme involved in nucleoside metabolism. In particular, TP is known to be essential for the activation of capecitabine from the intermediate form 5′-deoxy-5-fluorouridine (5′-DFUR) to the active form 5-fluorouracil (5-FU). Experimental studies showed that 5′-DFUR is much more active in TP-transfected cells than in mock-transfected cells (Patterson *et al*, 1995; Evrard *et al*, 1999). It is also true that 5′-DFUR is more effective for TP-overexpressing tumour xenografts than for tumour xenografts expressing normal or low levels of TP (Morita *et al*, 2001; Ishikawa *et al*, 1998). Furthermore, several preliminary studies also confirmed that TP expression in tumour cells was a predictive factor for favourable prognosis in cancer patients treated with 5′-DFUR (Yamamoto *et al*, 1996; Ishii *et al*, 1996; Koizumi *et al*, 1999; Nishimura *et al*, 2002). In primary breast cancer, an analysis of the relationship between TP expression and the therapeutic effect of 5′-DFUR as a retrospective study in a prospective clinical randomised study has recently been reported, where patients who received no systemic adjuvant treatment were compared with those who received treatment with 5′-DFUR alone. It concluded that TP is a promising marker for predicting the survival benefit from 5′-DFUR treatment in early breast cancer patients (Tominaga *et al*, 2002).

On the other hand, a hypothesis that TP modulation could enhance the therapeutic activity of 5′-DFUR/capecitabine has been tested at the experimental level. In various types of tumour xenograft models, the combination of capecitabine and various TP modulating chemotherapeutic agents achieved synergistic effects (Sawada *et al*, 1998; Fujimoto-Ouchi *et al*, 2002). Differences in the duration between the induction chemotherapy, with respect to TP modulation, and capecitabine treatment elicited different tumour responses, indicating that TP modulation is time dependent (Fujimoto-Ouchi *et al*, 2001) and that the timing of capecitabine treatment after the initial chemotherapy is important. In a clinical situation, it was demonstrated that therapy with capecitabine plus TXT achieved a significantly higher response and longer time to progression (TTP) than TXT therapy alone in the first-line treatment of metastatic breast cancer patients (O'Shaughnessy *et al*, 2002). This clinical finding would reflect on the basic hypothesis that TXT sensitises tumours to the effect of capecitabine. This suggests the importance of considering TP modulation from the point of sensitising breast cancer tumours to 5-FU derivatives such as capecitabine and 5′-DFUR, because the likelihood of their efficacy might be increased for TP upregulated tumours.

Issues related to TP modulation in human tumour tissues, however, are still largely unknown. Very few papers have touched on this crucial question. Thus, in the present study, we examined TP expression prior to and after the administration of chemotherapy in a neoadjuvant setting of primary breast cancer treatment. We will demonstrate that TP expression is modulated significantly by certain chemotherapies in a defined patient population.

## MATERIALS AND METHODS

### Patient characteristics

Between January 1, 1998 and December 30, 2002, women at the Tokyo Metropolitan Komagome Hospital and the National Kyushu Cancer Hospital who had primary, palpable, operable breast cancer (T2-4N0-1M0, according to the tumour, node, metastasis staging system) were included in this study. All patients were diagnosed by core needle biopsy or excisional biopsy prior to starting chemotherapy, and all patients were informed about the investigational nature of the study, which had been approved by the institutional review board. Written informed consent was obtained from each woman before entering her into the trial. All patients received either partial mastectomy or modified radical mastectomy with full dissection of axillary nodes after the treatment by neoadjuvant chemotherapy. Both biopsied and surgically resected samples were sufficient for accurate histological diagnosis and measurement of biomarkers.

### Treatment regimens

Patients were treated with anthracycline-containing regimens or a taxane-containing regimen. The anthracycline-containing regimens consisted of adriamycin (ADR) and cyclophosphamide (CPA), (AC); epirubicin (EPI) and CPA (EC) or 5-FU, ADR, and CPA (FAC); and 5-FU, EPI, and CPA (FEC). Patients were given chemotherapy every 21 days with either the AC (ADR 50 mg m^−2^ and CPA 500 mg m^−2^), EC (EPI 75 mg m^−2^ and CPA 600 mg m^−2^), FAC (5-FU 500 mg m^−2^, ADR 50 mg m^−2^, and CPA 500 mg m^−2^), and FEC (5-FU 500 mg m^−2^, EPI 100 mg m^−2^, and CPA 500 mg m^−2^) or the TXT-containing regimen (FEC followed by TXT 75 mg m^−2^ or TXT 60 mg m^−2^).

### Efficacy assessment

Responses of the primary tumours to each chemotherapy regimen were evaluated according to the criteria established by the Japanese Breast Cancer Society (The Japanese Breast Cancer Society, 2000), which are essentially the same as those of the World Health Organization. A complete response (CR) is defined as the disappearance of tumour; partial response (PR) refers to a decrease in tumour size of 50% or more; no change (NC) indicates a decrease in tumour size of 50% or less or an increase of tumour size by less than 25%; and progressive disease (PD) indicates an increase in tumour size of 25% or more.

The grading of the pathological efficiency of chemotherapy, which was evaluated microscopically by a skilled pathologist, was also categorised according to the criteria established by the Japanese Breast Cancer Society (The Japanese Breast Cancer Society, 2000). The three grades are defined as follows: Grade 3 is the complete disappearance of variable cancer cells on the examined specimens; Grade 2, the apparent degeneration of two out of three or more of the population of observed cancer cells; Grade 1, the presence of degenerated cells in less than two out of three of examined tumour cells; and Grade 0, the presence of no degenerative cancer cells on specimens.

### Immunohistochemical assessment

All samples were retrospectively processed with haematoxylin–eosin staining, negative control staining, and immunostaining for TP in our laboratory. Thymidine phosphorylase antibody was obtained from Roche Diagnostics (Basel, Switzerland), and the method for immunohistochemistry followed the protocol given in the immunohistochemistry kit ‘Anti-TP Antibody, Formalin-Grade’ (Roche Diagnostics Corporation, USA). The TP-stained slides were assessed for tumour cells and stromal cells according to the criteria defined in the kit. Staining intensities were scored as one of the four grades 0, 1+, 2+, and 3+, and staining patterns were scored as one of the five grades 0, 1+, 2+, 3+, and 4+.

Oestrogen receptor (ER) status progesterone receptor (PR) was also determined by an immunohistochemical method as described previously (Saji *et al*, 2002). Tumours containing 10% or more receptor-positive cells were scored as being receptor-positive.

### Statistical methods

All patients with tissue staining data were included in the analysis. The statistical analyses for the TP-immunostained preparations were conducted as follows. The four grades of staining intensities were scored as 0, 1, 2, and 3. Similarly, the five grades of staining patterns were scored as 0, 1, 2, 3, and 4. Thymidine phosphorylase up- or down-regulation was evaluated as the difference between the sample score after chemotherapy minus the sample score prior to chemotherapy for each patient. Samples with score differences greater than 1 were evaluated as ‘upregulated’, and less than −1 as ‘downregulated.’ Score differences in the range between −1 and 1 were evaluated as ‘no change.’ Scores of staining intensities and staining patterns were analysed, and the summation of staining intensity and pattern scores were also analysed. After checking the distribution of the score differences, the *t*-test was used to compare the means.

For the contingency tables, Fisher's exact test was used to assess the potential different distribution. To relate the score differences with the treatment groups, we used the Mantel–Haenszel test for contingency tables and the *t*-test to compare the means. Since the known prognostic factors such as tumour size were distributed differently in each treatment group, tumour size was used as a stratified factor for both the Mantel–Haenszel and *t*-test. Bonferroni's correction was applied to adjust the *P*-values of the pairwise comparisons between each treatment group.

All analyses were carried out by using SAS 8.2, and alpha was set at 0.05.

## RESULTS

### Patient characteristics

A total of 92 patients were enrolled in this study. All the 92 patients were eligible and provided tissue staining results. The patient characteristics are shown in Table 1

**Table 1:** Patients' characteristics and overall response rate

			**Regimen (%)**		
**Characteristics**	***n***	**AC/EC**	**FAC/FEC**	**TXT**	*P*-value*
*Menopausal status*					
Pre	46	5 (10.9)	27 (58.7)	14 (30.4)	
Post	46	9 (19.6)	31 (67.4)	6 (13.0)	0.107
					
*Tumour size*					
−3.0 cm	11	0 (0.0)	2 (18.2)	9 (81.8)	
3.1 cm-	81	14 (17.3)	56 (69.1)	11 (13.6)	<0.001
					
*Number of nodes involved*					
0	19	4 (21.1)	5 (26.3)	10 (52.6)	
1–3	18	2 (11.1)	9 (50.0)	7 (38.9)	
4–	55	8 (14.6)	44 (80.0)	3 (5.4)	<0.001
					
*Oestrogen receptor*					
+	59	9 (15.3)	35 (59.3)	15 (25.4)	
−	33	5 (15.2)	23 (69.7)	5 (15.1)	0.571
					
*Progesterone receptor*					
+	39	6 (15.4)	26 (66.7)	7 (17.9)	
−	53	8 (15.1)	32 (60.4)	13 (24.5)	0.789
					
Cycle (median)	92	2–4 (4.0)	2–6 (3.0)	7–8 (8.0)	—
Response rate	92	50.0%	41.4%	70.0%	—
(95% CI)		(23.0–77.0)	(28.6–55.1)	(45.7–88.1)	—

AC=adriamycin (ADR) and cyclophosphamide (CPA); EC=epirubicin (EPI) and CPA; FAC=5-fluorouracil (5-FU), ADR, and CPA; FEC=5-FU, EPI, and CPA, TXT=docetaxel-containing regimen, CI=confidence interval,

* Fisher's exact test.

. Imbalances were observed for tumour size and number of patients, *n*, between the treatment groups, which would not affect the results of the present study, because no correlation was observed with TP regulation as reported below. At initial diagnosis, the average age of the women in this study was 51 years (range, 28–74 years). With respect to tumour size, those of 11 patients were less than 3.0 cm and those of 81 patients were greater than 3.1 cm. In all, 79% of patients had positive nodal status and 64.1% of patients had oestrogen-receptor-positive tumours.

Among the patients, 14 were treated with AC or EC, 58 were treated with FAC or FEC, and 20 were treated with the TXT-containing regimen.

### Thymidine phosphorylase immunohistochemistry

**Figure 1:**
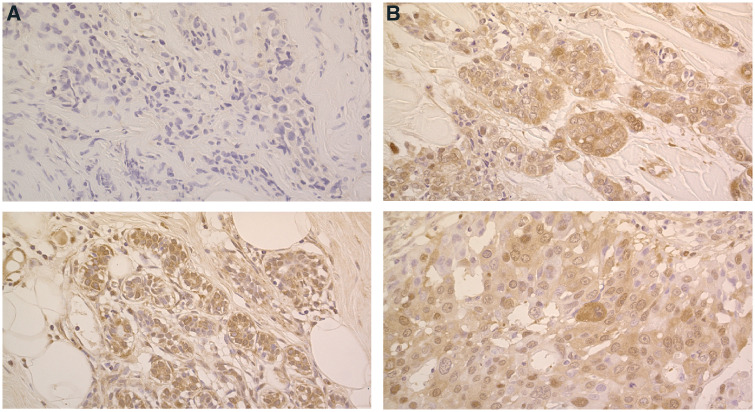
TP expression status of pre- and post-treatment. (**A**) An invasive ductal carcinoma: TP expression was upregulated remarkably by the treatment with FEC (5-FU, epirubicin, and cyclophosphamide) followed by docetaxel. Tumour TP score: pretreatment; 0 (upper), post-treatment; 7 (bottom), yielding a score difference of 7. The treatment achieved PR. (**B**) An invasive ductal carcinoma; TP expression was not changed remarkably by the treatment with FEC followed by docetaxel. Tumour TP score: pretreatment; 6 (upper), post-treatment; 5 (bottom), yielding a score difference of −1. The treatment achieved PR.

We used the difference in each patient's tissue staining scores before and after chemotherapy to assess TP up- or down-regulation (Figure 1

). Thymidine phosphorylase scores, staining intensities, and staining patterns from both tumour cells and stromal cells were available. No correlations were observed between the tumour and stromal scores. TP changes were seen in response to chemotherapy; TP levels in tumour and stromal cells were upregulated in 50 patients (54.4%) and 30 patients (32.6%), and downregulated in 15 patients (16.3%) and 29 patients (31.5%), respectively.

Table 2

**Table 2:** Association of (**A**) tumour TP changes and (**B**) stromal TP changes with patients' characteristics

**(A) Tumour TP changes**				
	**Tumour TP**			
	**Up (%)**	**NC (%)**	**Down (%)**	*P*-value*
*Menopausal status*				
Pre	29 (63.0)	9 (19.6)	8 (17.4)	
Post	21 (45.7)	18 (39.1)	7 (15.2)	0.122
				
*Tumour size*				
Median (range)	6.2 (1.5–18.0)			
−3.0 cm	6 (54.6)	2 (18.2)	3 (27.3)	
3.1 cm –	44 (54.3)	25 (30.9)	12 (14.8)	0.456
				
*Number of nodes involved*				
0	13 (68.4)	5 (26.3)	1 (5.3)	
1–3	9 (50.0)	5 (27.8)	4 (22.2)	
4–	28 (50.9)	17 (30.9)	10 (18.2)	0.578
				
*Oestrogen receptor*				
Positive	36 (61.0)	16 (27.1)	7 (11.9)	
Negative	14 (42.4)	11 (33.3)	8 (24.2)	0.157
				
**(B) Stromal TP changes**				
	**Stromal TP**			
	**Up (%)**	**NC (%)**	**Down (%)**	*P*-value*
*Menopausal status*				
Pre	15 (32.6)	16 (34.8)	15 (32.6)	
Post	15 (32.6)	17 (37.0)	14 (30.4)	1.000
				
Tumour size				
Median (range)	6.2 (1.5–18.0)			
−3.0 cm	0 (0.0)	7 (63.6)	4 (36.4)	
3.1 cm–	30 (37.0)	26 (32.1)	25 (30.9)	0.020
				
*Number of nodes involved*				
0	10 (52.6)	3 (15.8)	6 (31.6)	
1–3	4 (22.2)	9 (50.0)	5 (27.8)	
4–	16 (29.1)	21 (38.2)	18 (32.7)	0.173
				
*Oestrogen receptor*				
Positive	21 (35.6)	20 (33.9)	18 (30.5)	
Negative	9 (27.3)	13 (39.4)	11 (33.3)	0.736

TP=thymidine phosphorylase; Up=upregulated; NC=no change; Down=downregulated;

* Fisher's exact test.

shows the correlation between TP changes and patients' characteristics (Table 2A: tumour, 2B: stroma, respectively). An association between them was seen only in tumour size for stromal TP (*P=*0.020). On the other hand, there were no significant differences for relationships for the number of nodes involved, ER status, or menopausal status.

Table 3

**Table 3:** Tumour TP changes by each regimen

**Regimen**	***n***	**Gain in TP score (mean)**	**Up (%)**	**NC (%)**	**Down (%)**
*AC/EC*					
Tumour	14	4.3	13 (92.9)	1 (7.1)	0 (0.0)
Stroma	14	3.6	12 (85.7)	2 (14.3)	0 (0.0)
					
*FAC/FEC*					
Tumour	58	0.7	24 (41.4)	22 (37.9)	12 (20.7)
Stroma	58	−0.9	10 (17.2)	25 (43.1)	23 (39.7)
					
*TXT-containing regimen*					
Tumour	20	1.8	13 (65.0)	4 (20.0)	3 (15.0)
Stroma	20	0.0	8 (40.0)	6 (30.0)	6 (30.0)
					
Total					
Tumour	92	—	50 (54.4)	27 (29.3)	15 (16.3)
Stroma	92	—	30 (32.6)	33 (35.9)	29 (31.5)
					
		**Tumour**		**Stroma**	
**Regimen compared**		***t*-test**	**M–H**	***t*-test**	**M–H**
AC/EC *vs* FAC/FEC		<0.0001	0.0114	<0.0001	<0.0001
FAC/FEC *vs* TXT		0.2287	0.5700	0.0580	0.0021
AC/EC *vs* TXT		0.1527	0.5616	0.0339	0.7773

Up=upregulated; NC=no change; Down=downregulated; AC=adriamycin (ADR) and cyclophosphamide (CPA); EC=epirubicin (EPI) and CPA; FAC=5-fluorouracil (5-FU), ADR, and CPA; FEC=5-FU, EPI, and CPA; P-values with Bonferroni's correction, adjusted by tumour size; M–H=Mantel–Haenszel test.

shows the relation between TP changes and treatment groups. TP changes were lowest in the FAC/FEC group and highest in the AC/EC group. Adjusted *P*-values of pairwise comparisons by Bonferroni's correction suggest that the TP score changes in the FAC/FEC group are significantly different from those in the AC/EC group (tumour: *P*=0.0001, stromal: *P*=0.0001). Nevertheless, no association was observed between scores of tumour and stroma, and the association with treatment regimen was similar for both tumour and stroma.

In the AC or EC group, TP was upregulated in the tumour and stromal cells of 92.9 and 85.7% of patients, respectively; however, TP was not downregulated in any patient. In the FAC or FEC group, tumour TP was upregulated in 41.4% of patients and downregulated in 20.7%. In the TXT-containing regimen, tumour TP was upregulated in 65.0% of patients and downregulated in 15.0%.

### Clinical response rates

Of the 92 patients available for analysis, an overall response rate (ORR) of of 50.0%. (95% confidence interval (CI): 23.0–77.0%) was achieved by patients who were treated with AC or EC, an ORR of 41.4% (95% CI: 28.6–55.1%) by the patients treated with FAC or FEC, and an ORR of 70.0% (95% CI: 45.7–88.1%) by those patients given the TXT-containing regimen, as shown in Table 1.

The relationship between ORR and TP status is shown in Table 4

**Table 4:** Relationship between TP changes and response

	***n***	**Up (%)**	**NC (%)**	**Down (%)**	*P*-value*
*Tumour*					
Responder	45	24 (53.3)	16 (35.6)	5 (11.1)	
Nonresponder	47	26 (55.3)	11 (23.4)	10 (21.3)	0.383
					
*Stroma*					
Responder	45	14 (31.1)	14 (31.1)	17 (37.8)	
Nonresponder	47	16 (34.0)	19 (40.5)	12 (25.5)	0.461

Up=upregulated; NC=no change; Down=downregulated;

* Mantel–Haenszel test adjusted by tumour size.

. There was no correlation observed between clinical response and TP status, for either tumour or stromal cells (*P*=0.383 and *P*=0.461, respectively).

### Pathological response rate

Of the 87 patients available for analysis, a grade 2 response was achieved by 14.3%. of patients who were treated with AC or EC (95% CI: 1.78–42.8%), 12.1% of those treated with FAC or FEC (95% CI: 4.99–23.3%), and 6.7% of those treated with the TXT-containing regimen (95% CI: 0.17–32.0%). Overall, a grade 2 response of 11.5% (95% CI: 5.65–20.1%) was seen in this study. There was no significant correlation between the pathological responses of grade 2 and TP changes in both tumour and stromal cells (*P*=0.600 and *P*=0.273, respectively).

## DISCUSSION

Although the predictive value of TP expression in tumour tissues has been studied extensively for 5-FU or 5-FU-containing treatments, there is still little known about changes in TP levels in human tumours in response to chemotherapy. In this study, we showed that TP expression is often enhanced in primary breast tumours in response to neoadjuvant chemotherapy. In particular, we found that TP was frequently upregulated in response to treatment by an EC/AC- or TXT-containing regimen. These results seem to be compatible with the data for human cancer xenograft experiments where taxanes, CPA, and mitomycin-C showed the potent ability to upregulate TP (Sawada *et al*, 1998; Endo *et al*, 1999). TXT also caused TP upregulation as a neoadjuvant in advanced breast cancer patients (Kurosumi *et al*, 2000), a result that also seems to be compatible with the clinical data. Thymidine phosphorylase in tumour cells tended to be co-upregulated with TP in tumour-associated stromal cells such as macrophages, indicating a possible role for microenvironmental factors in this response. In previous studies looking at correlations between TP and various immune mediators in the human breast tumour microenvironment, TP expression was associated significantly with expressions of TNF-*α* (Leek *et al*, 1998), IL-1*α* (Eda *et al*, 1993), and monocyte chemoattractant protein (MCP)-1 (Saji *et al*, 2002). From the molecular profile, it is known that multiple copies of potential Sp-1 binding sites are clustered upstream of the start site for the initiation of TP transcription (Zhu *et al*, 2002). Therefore, it is possible that TP upregulation would be triggered by increases in the intratumoural concentrations of these immune mediators in response to chemotherapy. As chemotherapy causes massive damage in tumour cells, the immune cells, especially macrophages, are activated to eliminate the damaged cells. In this process, it is estimated that large amounts of chemical immune mediators are produced by tumour-associated macrophages in the tumour microenvironment. Since hypoxia and hypoglucose are also characterised as stimuli of TP expression (Griffiths *et al*, 1997), these physical factors might help to enhance TP expression in association with local hyper-cytokinaemia.

For those patients treated with FAC or FEC, the 5-FU-containing regimens, we found no increased frequency of TP upregulation after chemotherapy. There are at least two possible explanations for this phenomenon. Firstly, the high concentration of 5-FU might downregulate TP expression. It is known that high concentrations of pyrimidine substrate change or downregulate the expression levels of nucleoside metabolism enzymes. There are few reports investigating the effect of high concentrations of 5-FU on TP; however, this mechanism is likely to be involved.

Secondarily, 5-FU might selectively kill or suppress TP-overexpressing cells. Many basic and clinical studies have indicated that 5-FU-contaning regimens are more effective for TP-overexpressing tumour cells as compared with TP-less-expressing tumour cells (Fox *et al*, 1997; Evrard *et al*, 1999; Gasparini *et al*, 1999; Morita *et al*, 2001; Yang *et al*, 2002). Therefore, these two scenarios should be further studied. Thymidine phosphorylase is stress-induced and, basically, TP is shown to be an enzyme that contributes to cell survival, because 2-DDR, a metabolite of thymidine via TP, induces neovascularisation and contributes to antiapoptosis (Haraguchi *et al*, 1994). After exposure to chemotherapy, TP might also function as mechanism for survival by the tumour cells. Based upon this hypothesis, a sequential treatment consisting of TP-upregulating chemotherapy followed by TP-dependent chemotherapy, such as by capecitabine, might be a reasonable therapeutic approach. In fact, the combination treatment with taxane and capecitabine showed a synergistic effect in animal experiments (Sawada *et al*, 1998) and induced a significant improvement in the survival of metastatic breast cancer patients (O'Shaughnessy *et al*, 2002). Therefore, the examination of TP expression in detail might provide various ideas to consider about optimal combinations in dosage and timing between capecitabine and other chemotherapeutic drugs. For example, a TC or TAC regimen might be promising to induce maximal TP expression. Furthermore, in cases where TP is not upregulated after the initial chemotherapy, the subsequent capecitabine monotherapy might not be effective.

In conclusion, TP is frequently up- or down-regulated after EC/AC- or taxane-containing chemotherapy in primary breast tumour tissues. The upregulated levels of TP are less for 5-FU-containing regimens. Thymidine phosphorylase is indeed upregulated by several anticancer drugs in human breast cancer cells, including both tumour and stromal cells; however, there are variations in the level. Thus, it is important to consider an individual strategy for sensitisation of breast tumour tissues to 5-FU by chemotherapy through TP.
